# Exploring the Potential of Proteome Analysis as a Promising Tool for Evaluation of Sudden Cardiac Death (SCD) in Forensic Settings: A Literature Review

**DOI:** 10.3390/ijms241814351

**Published:** 2023-09-20

**Authors:** Matteo Antonio Sacco, Saverio Gualtieri, Luca Calanna, Pietrantonio Ricci, Isabella Aquila

**Affiliations:** Institute of Legal Medicine, Department of Medical and Surgical Sciences, “Magna Graecia” University, 88100 Catanzaro, Italy; matteoantoniosacco@gmail.com (M.A.S.); saveriogualtieri@icloud.com (S.G.); luca.calanna@studenti.unicz.it (L.C.); ricci@unicz.it (P.R.)

**Keywords:** sudden cardiac death, forensic sciences, proteomics

## Abstract

Sudden cardiac death (SCD) represents a global emergency, with a high number of cases affecting all age groups every year. The prevention of these fatal events requires an accurate knowledge of etiology and pathogenesis, which can vary. Autopsy is an indispensable tool in cases of SCD for diagnostic purposes, as well as for judicial and preventive purposes for family members. Despite the completion of all routine post-mortem investigations, it is often complicated for the forensic pathologist to define the triggering cause of these events. The study of the proteome is proving to be extremely promising in the field of human cardiovascular disease. This paper aims to offer a literature review on the study of the proteome in post-mortem cadaveric biological samples obtained from SCD cases. The aim of this work is to outline the state of the art of the scientific advances that protein analysis can offer in the diagnosis of SCD and the limits that various studies have traced up to now. In conclusion, the work defines the future perspectives of this field in SCD, suggesting strategies to overcome the reported limits and improve the diagnostics of these events.

## 1. Introduction

### 1.1. Epidemiology of Sudden Cardiac Deaths

Sudden cardiac death (SCD) is defined as an unexpected death occurring within one hour of symptom onset or within 24 h in apparently healthy individuals [1]. SCD is a global health emergency [2]. Statistics show that cardiovascular deaths account for a third of all deaths globally and are the primary cause of premature mortality [3]. It has also been estimated that more than 3 million people per year die from SCD. The main cause of sudden cardiac death is attributable to fatal arrhythmogenic mechanisms due to coronary heart disease, especially in subjects over 50 years old. In Sweden, the incidence of sudden cardiac death in individuals aged 35–64 without previous cardiovascular disease is equal to 65/100,000 cases in men and 12/100,000 cases in women [4]. Approximately 85% of sudden deaths can therefore be associated with cardiac causes, responsible for 30–200 deaths per 100,000 each year in the western territory. In the United States, a total of 300,000 deaths per year from SCD have been calculated, and in the United Kingdom, a total of 60,000 deaths per year [5,6]. Epidemiological data demonstrate that adequate prevention of these fatal events is essential. Prevention strategies must include an accurate study of post-mortem SCD episodes in order to analyze the pathogenetic and molecular mechanisms that lead to these events.

### 1.2. The Role of Autopsy in the Diagnosis of SCD

The gold standard for determining the cause of death is certainly autopsy [7]. In these cases, the forensic pathologist analyzes the external signs, such as hypostases on the head and neck, and internal signs with gross and microscopic examination of the heart. The gross analysis of the myocardium should include the evaluation of any signs of ischemia, the careful study of the coronary tree, and the visualization of any congenital structural anomalies. The microscopic study can allow us to better visualize the degree of coronary stenosis and to examine the signs of ischemia with accumulation of granulocyte infiltrates, “wavy fibers” and possible necrosis [8,9]. Signs related to SCD cases may also include severe blood congestion and pulmonary edema. Despite accurate post-mortem investigations and the exclusion of the intake of exogenous substances with a toxicological examination, it is often very complex to determine the cause of SCD [7,10,11]. The analysis of any exogenous substances, such as illicit drugs, alcohol and psychotropic drugs, plays a fundamental role in the analysis of SCD cases, and the search for these substances with first-level and second-level toxicology investigations is mandatory in order to exclude potential intoxication, even if macroscopic or microscopic cardiac anomalies were found. This investigation should be carried out on at least two cadaveric biological matrices, such as peripheral blood and urine [7]. In the forensic setting, it has been estimated that approximately 10% of these deaths remain unexplained despite autopsy and that up to 5% of the deaths remain unexplained despite carrying out all routine post-mortem investigations, including toxicological investigations [12,13]. Of the autopsied cases over the age of 35, the literature shows that more than 30% of the cases remain unexplained despite all traditional forensic investigations [14,15,16]. In fact, in many cases, autopsy shows the presence of coronary artery disease of various degrees without evident signs of myocardial ischemia, both macroscopic and microscopic. Furthermore, the presence of coronary plaques is contextually visible at autopsy even in cases not attributable to SCD; therefore, it is often very difficult to define their role in the genesis of death. In addition to coronary atherosclerosis, it is evident that there are alternative pathogenetic mechanisms capable of causing fatal arrhythmias and death. The study of these mechanisms, especially in cases of so-called negative autopsy, must necessarily include a molecular investigation.

### 1.3. Etiology of Sudden Cardiac Deaths

**Figure 1:**
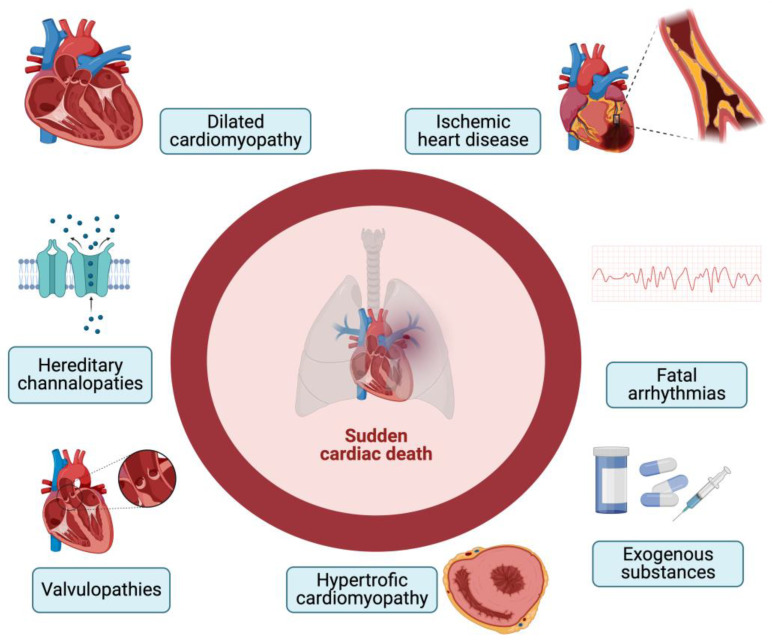
Causes of SCD (created with BioRender.com).

The causes of SCD can be diverse. Among these, we cite structural causes such as cardiac remodeling, which arises in various heart diseases such as hypertrophic cardiomyopathy, dilated cardiomyopathy, and chronic heart failure [17,18] (Figure 1). There are also coronary-related causes that include coronary spasm, atherosclerosis up to ischemia, and myocardial infarction. It should also be considered that a proportion of SCDs occurs in the absence of ischemic alterations in subjects with anomalies of the electrical conduction system and that exogenous substances are triggered in some SCD cases [19]. The differential diagnosis of these cases with standard post-mortem autopsy and histopathological findings is extremely complex. Kakimoto et al. demonstrated that, from a histological point of view, cases of sudden cardiac death from acquired or compensated hypertrophy are superimposable, as they both present high levels of muscle hypertrophy and moderate myocardial fibrosis [20]. Furthermore, histopathological analysis very often shows an interindividual variability related to the experience of the operator and to the interpretation of the microscopic images; therefore, it is not always decisive in diagnosing the exact etiology. This limit can be important not only for epidemiological and preventive purposes but also in cases of judicial interest, in which it is essential to know with certainty the cause of death and any legal responsibilities connected to it [21].

### 1.4. The Potential of the Proteome in Diagnosing Sudden Cardiac Deaths

The proteome represents the set of proteins produced by a cell or an organism. The proteome is known to be much larger and more complex than the genome, especially in eukaryotes, due to the high number of proteins and functional interactions. The study of the functions of the proteome and its regulatory mechanisms is called proteomics. This is a discipline that today makes use of functional studies and quantitative analyses of proteins for the study of protein profiles of cell lines, tumor tissues, and disease analyses. The first-choice method in a proteomics study is represented by two-dimensional gel electrophoresis with SDS-PAGE, a technique that allows the separation of proteins according to the isoelectric point and molecular weight and subsequently the use of fluorescent colors for the recognition of proteins. Another fundamental technique in the study of proteomics is mass spectrometry. With this method, the peptides are brought into the gaseous phase and ionized, and the mass/charge ratio is measured. With spectrometry, it is possible to apply techniques such as peptide mass fingerprinting, up to splitting a protein into protein segments and comparing the masses of the peptides with reference databases. Proteomics is a discipline that has seen a great evolution by expanding towards new technologies with greater possibilities for protein profiling and discovery of diagnostic biomarkers [22]. The interaction between proteins, metabolites, and interactors can modulate a large number of cellular processes, including metabolic pathways, growth signals, and cellular stress [23,24]. Therefore, the study of proteins and the integrated analysis of data with new proteomics strategies can offer a molecular view of various physiological and pathological conditions. In recent years, several works have investigated the role of the proteome in the development of various pathologies, especially in cardiology. In the face of numerous clinical studies, the study of the proteome is progressively advancing in the forensic setting [25]. Despite its incredible potential, to date, the role of proteomics in diagnosing the etiopathogenetic mechanisms underlying SCD remains poorly understood. The present review aims to evaluate the possible application of the study of protein analysis in the identification of the causes of SCD in forensic settings.

## 2. Materials and Methods

A literature review of the past 10 years (from 1 January 2013 to 31 August 2023) was performed using the PubMed NCBI and Scopus Embase search engines. Research studies describing experimental analyses carried out on post-mortem biological samples were investigated using the following keywords: proteomic OR proteome OR protein AND sudden cardiac death AND forensic AND autopsy. Original works in the English language were examined. Related bibliographic entries were also evaluated. Works in other languages and papers not related to protein analysis but focused exclusively on histopathological methods were excluded.

## 3. Results and Discussion

**Figure 2:**
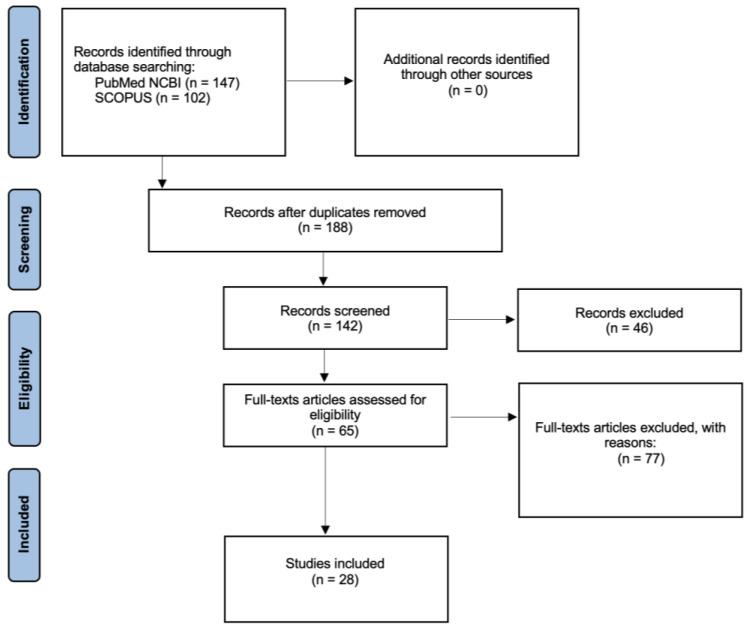
Search method using the PRISMA flowchart.

A total of 28 studies were included in this review (Figure 2).

### 3.1. Analysis of Hypertrophic Cardiomyopathy

Cardiac hypertrophy is an important risk factor associated with SCD [26]. The pathophysiology of cardiac hypertrophy generally includes mechanisms of hypertension and an increase in cardiac volume until it evolves, due to structural remodeling, into heart failure with a considerable increase in the risk of SCD [27]. Several clinical studies about proteomics have focused on the contribution that the study of the proteome can offer in cases of myocardial hypertrophy. In particular, in cases of cardiac death associated with myocardial hypertrophy, it has been demonstrated that there is an increase in the levels of sarcomeric proteins such as MYH7 and MYL3. This increase was evaluated in cases of acquired hypertrophic cardiomyopathy (i.e., hypertrophy accompanying risk factors such as obesity, hypertension, coronary artery disease), the upregulation of which can lead to an increased risk of SCD. The analysis was performed on myocardial tissue samples subjected to quantitative proteomic analysis with LC-MS on three cadavers. Despite the limitation of the smallness of the sample, the study of myocardial samples from cadavers proves useful in furthering the diagnosis of deaths related to acquired cardiac hypertrophy [20].

### 3.2. Analysis of Mechanisms of Coronary Spasm

Coronary spasm refers to intense vasoconstriction of the coronary arteries, resulting in subtotal vascular occlusion [28]. This pathology, already described in the 1950s by Prinzmetal et al., is considered fundamental for causing fatal pathogenetic mechanisms underlying SCD, such as acute coronary syndrome and ischemic myocardial disease. The mechanisms that lead to SCD from coronary atherosclerosis include transient spasm with coronary stenosis and hypoflow of blood to the heart [29,30,31,32]. In a recent work, Lin et al. studied the mechanisms of coronary artery spasm in rabbit models by evaluating SELENBP1 and VCL as candidate biomarkers. The authors used pituitrin injection to induce coronary spasm in animals and subsequently performed quantitative serum proteomics with parallel reaction monitoring/mass spectrometry-based targeted proteomics, demonstrating that SLENBP1 and VCL can be candidate biomarkers in both animal and human models. In particular, the authors examined the serum levels of a sample of 12 cadavers who died of SCD subjected to autopsy, detecting a diagnostic specificity of SELENBP1 and VCL (100.0%) higher than traditional markers, such as cTnI and CK-MB. These markers can therefore prove to be very promising for diagnosing coronary spasm processes, although further studies are needed, especially on cadavers, to validate their application for diagnostic purposes [33].

### 3.3. Analysis of Mechanisms of Atherosclerosis

Atherosclerosis is considered a complex inflammatory disease of a chronic nature that affects the vessel wall, with particular interest at the level of the coronary tree. This phenomenon affects various inflammatory cells, including granulocytes and macrophages, with complex cellular signaling. The analysis of the atherosclerosis mechanisms and of the related inflammatory cascade is now the subject of several post-mortem studies [34,35,36].

Dai et al. subjected three cases of SCD versus three control cases to quantitative proteomic analysis [14]. The cases analyzed showed coronary artery disease with anterior descending artery stenosis in the presence of plaques and luminal stenosis greater than 50%. Specimens submitted for analysis consisted of coronary artery specimens extracted from autopsies and subsequently subjected to protein extraction, trypsin digestion, and LC-MS analysis. A significant increase in proteins belonging to the cathepsin family was observed, i.e., a family of hydrolytic prostheses expressed in lysosomes, involved in inflammation and in lysosomal pathways. These families are fundamental in the mechanisms of autophagy, cellular stress signaling, and lysosome-dependent cell death. These proteins are also known for inducing atherosclerosis; in fact, several studies suggest that the amount of cathepsins produced increases with the mechanisms of atherosclerosis and plaque rupture. The authors therefore hypothesized that cathepsin c (CTSC) may be a key target for regulating Mq macrophage polarization in SCD via the p38/MAPK pathway [14].

In another study, the role of activating transcription factor 3 (ATF3), i.e., a protein that acts as a transcription factor related to coronary atherosclerosis, was evaluated. Several studies have demonstrated the role that ATF3 plays in various processes, including apoptosis, oxidative stress, inflammation, and cellular stress [37]. In particular, this protein is involved in several pathological contexts, such as extracellular matrix dysfunction and the development of atherosclerosis, although it is not clear whether it is a promoter or an inhibitor of atherosclerosis mechanisms. In one study, Peng et al. evaluated 68 coronary artery samples, of which 36 were cases of sudden death associated with plaque rupture with intra-plaque hemorrhage, while the remaining were the control group, i.e., they had coronary atherosclerosis but died of other causes [38]. Protein expression was analyzed using Western blotting. The analysis showed that in control cases associated with stable coronary plaque, AFT3 levels were higher, proving that this protein could be related to the progression of atheromatous plaques, regulating their inflammatory response. The authors also evaluated the role of inflammatory factors with Western blot, i.e., TNF-alpha, IL-1B, CD-45, VCAM, and MMP-9, demonstrating a greater expression in the group of sudden deaths associated with plaque rupture. The study demonstrates how all these proteins could be evaluated as useful markers for the diagnosis of coronary plaque rupture, although standard investigation protocols are not yet known [38].

As we have seen, the evolution of the pathogenetic mechanisms that lead to plaque rupture from atherosclerosis is very difficult to study after death without appropriate molecular investigations. The mechanisms of atherosclerosis with coronary stenosis can result in so-called acute coronary syndrome (ACS) [39]. ACS, which includes unstable angina pectoris and acute myocardial infarction, is often difficult to investigate forensically. In fact, the autopsy examination, both in the gross examination and in the microscopic histological visualization, can determine the severity of the coronary stenosis, but it is known that the fatal coronary syndrome is not exclusively associated with a stenosis of the lumen but with the rupture or erosion of a plaque atherosclerotic with thrombus formation. Visualization of the coronary thrombosis site at autopsy is often complex. Furthermore, it must be considered that the pathogenesis does not include visible molecular mechanisms with inflammation. Pentraxin 3 is a protein that has been clinically investigated as a marker of ACS. The characteristic of this protein is that it is secreted after activation of the Toll-like receptor and secretion of proinflammatory cytokines, with very rapid induction. PTX3 is produced by macrophages and smooth muscle cells present at the level of the atherosclerotic lesion. Tojo et al. evaluated the cadaver plasma concentration of the PTX3 protein compared to the control group [40]. Analysis of PTX3 protein concentration in the acute coronary syndrome with coronary thrombosis group showed a concentration above 110.0 ng/mL. This concentration was lower in cases of coronary stenosis without thrombosis and in cases of myocardial rupture. These findings suggest that the protein increases in cases of high-risk plaque rupture. The analysis also revealed a higher concentration in cadaveric plasma than in living plasma, but without correlation with the PMI. The data therefore suggest that there is an immediate release of neutrophils into the blood at or shortly after death.

### 3.4. Analysis of Mechanisms of Myocardial Ischemia

**Figure 3:**
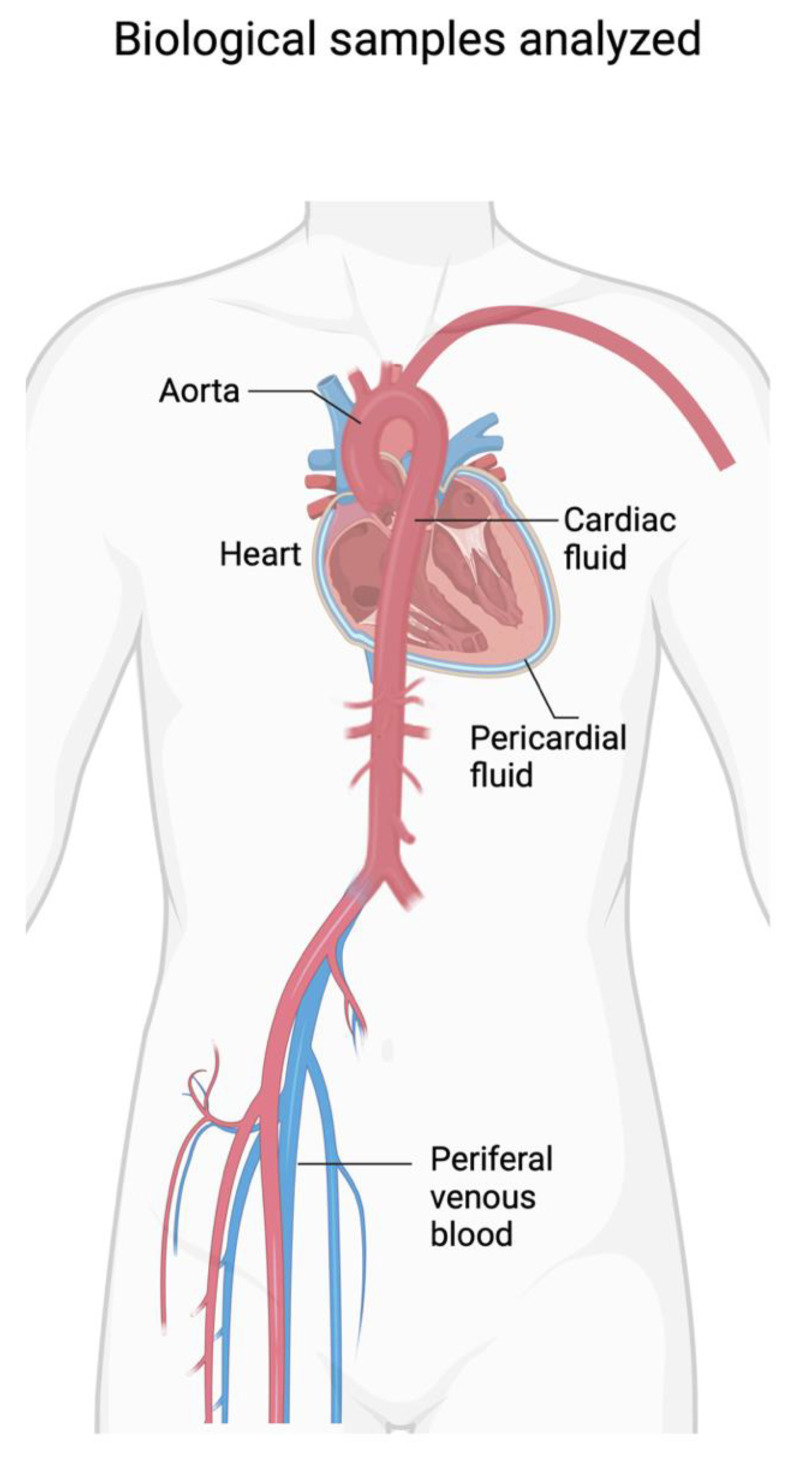
Biological samples analyzed in various studies on SCD (created with BioRender.com).

The most common cause of SCD is ischemic myocardial disease. According to the universal definition, the diagnosis of myocardial infarction requires the evaluation of signs and symptoms with a physical examination, the performance of the ECG examination, and the analysis of markers of cardiac damage. In the forensic field, the possibility of evaluating these data in post-mortem investigations is reduced, also considering that the microscopic infiltration by granulocytes in the myocardial tissue requires a timescale of several hours. To date, the gold standard for diagnosis, according to the European Society of Cardiology (ESC), is represented by the hs-cTnT marker [41]. Several studies have evaluated the diagnostic significance of the dosage of cardiac damage markers in cadaveric samples with different combinations (Figure 3).

According to Adams, the ideal marker of cardiac injury in the clinic should be present at high levels in myocardial tissue and should be released rapidly after ischemic insult, persisting in body fluids for several hours. From a clinical point of view, today cardiac troponin T (cTnT) and troponin I (cTnI) have replaced the CK-MB marker in the diagnosis of myocardial ischemia. However, the main limitation of these markers is associated with the low sensitivity at the time of clinical presentation due to a delayed rise of these proteins in the circulation. Today, the possibility of having highly sensitive markers has certainly improved diagnostic times in patients with symptoms.

Gonzalez-Herrera reported that the use of highly sensitive troponins is necessary, compared to troponin T or I alone, in the diagnosis of acute myocardial ischemia. In fact, compared to myocardial infarction, in myocardial ischemia the hypoxic insult acts for a shorter time on the myocardium in the absence of detectable necrosis; therefore, the diagnosis of these cases requires markers with greater stability [42]. The authors compared different biological fluids from cadavers, i.e., femoral venous blood and pericardial fluid, in a sample of 58 cases, of which 24 died from SCD, noting that only in the pericardial fluid there is a statistically significant difference in the levels of cTnT, with values ten times higher in pericardial fluid than in serum. The authors report that the pericardial fluid is a transudate that derives from the epicardium and that is well preserved by other substances and post-mortem processes. Another plausible explanation is that the pericardium is located near the myocardium with possible diffusion of the marker inside it, so it should be the ideal analysis matrix [42]. The authors conclude that a highly sensitive measurement of troponin T levels in the pericardial fluid should be a useful diagnostic test to define the cause of death.

Kutlu et al. evaluated a sample of various markers of cardiac damage in 35 post-mortem venous blood samples in SCD cases, namely hs-cTnT, NT-proBNP, H-FABP, pentraxin-3, copeptin, ischemic modified albumin (IMA), and PAPP-A, noting a consistent increase in the SCD grouo compared to the control [43]. The authors concluded that the combination of hs-cTnT markers, NT-proBNP, and pentraxin 3 offered the best sensitivity for diagnosing these cases. Another study evaluated the application of high-sensitivity cardiac troponin T (Hs-TnT) alone in 80 cases. The authors’ strategy was to compare the measurement of the marker in different post-mortem body fluids i.e., pericardial fluid, cardiac blood, and peripheral blood. The authors showed a significant correlation with better sensitivity (75%) and specificity (64%) using pericardial fluid as the reference biological fluid and using a cut-off level of 17.72 ng/mL [44].

Omalu et al. evaluated the levels of cTnI in cadaveric plasma in a sample of 52 subjects who died of type 3 myocardial infarction, detecting high levels (from 0.31 to greater than 4400 ng/mL) in 90% of the sample examined [45]. Beausire et al. evaluated the serum levels of the hs-TnT marker in a sample of 85 autopsies. The authors examined cases of ischemic heart characterized by coronary thrombosis or with signs of acute myocardial ischemia and cases of coronary stenosis greater than 75% [46]. Specimens consisted of analyses of peripheral venous blood collected from the femoral vein subjected to centrifugation for 15 min. Investigations demonstrated a significant nonlinear association between hs-TnT values and myocardial ischemia, concluding that the probability of cardiac death increases with increasing hs-TnT levels, reaching a maximum at 90 ng/L. The authors also considered that there is no correlation with attempted cardiopulmonary resuscitation maneuvers [46].

### 3.5. Analysis of Abnormalities of the Cardiac Electrical Conduction System

SCD can also occur due to mechanisms affecting the cardiac electrical conduction system in the context of various congenital syndromes, such as Brugada syndrome. In most cases, the electrical conduction system underlies fatal arrhythmogenic syndromes or hereditary channelopathies, QT-segment changes, and polymorphic catecholaminergic ventricular tachycardia [47].

In humans, the study of these mechanisms to date benefits from analyses such as molecular autopsy, i.e., the post-mortem search for genetic variants related to the alteration of proteins involved in cardiac electrical conduction or to specific congenital syndromes. The approach generally involves performing next-generation sequencing (WES) with the identification of altered gene and protein variants, classifiable as pathogenic, likely pathogenic or VUS—potentially pathogenic. This methodology is now widely described in the literature in various works, although our research did not reveal any proteomic approaches to human samples in the context of the study of electrical conduction anomalies.

In the animal field, however, two studies have examined the proteomic mechanisms in SCD-related ventricular tachycardia. The authors induced lethal ventricular tachycardia (LTVA) using aconitine in a group of rats (six rats in the test group and six in the control group). Subsequently, the myocardial proteome was analyzed using the LC-MS method. The results demonstrated upregulation of approximately 250 proteins and downregulation of approximately 200 other proteins in the LVTA group, suggesting an important disturbance in protein expression during the arrhythmogenic process. Investigations demonstrated important changes in proteins involved in energy metabolism, such as beta oxidation of fatty acids and tricarboxylic acid, inactivation of protein kinase C and NAPDH, alterations of the oxidase pathway, and alterations in proteins involved in intracellular calcium homeostasis, with increased oxidative stress and decreased ATP production [48]. In a similar study, the authors again, using an animal protocol with rats, induced LVTA by ligation of the coronary artery. The authors compared a sample of six cases with LVTA against a sample of six other cases in which death occurred with slower bradycardia mechanisms [49]. Proteomic analyses of myocardial samples revealed alterations in proteins involved in the mitochondrial respiration chain, including electron transport chain, oxidative phosphorylation, NADH dehydrogenase activity, oxidative phosphorylation, and citrate cycle. The results suggest that severe alterations of cellular metabolism occur in the mechanisms of fatal ventricular tachycardia, although to date no biomarkers for forensic use are known; to the best of our knowledge, there are no studies in this area on human samples after death.

### 3.6. The Role of Exogenous Substance Analysis

**Figure 4:**
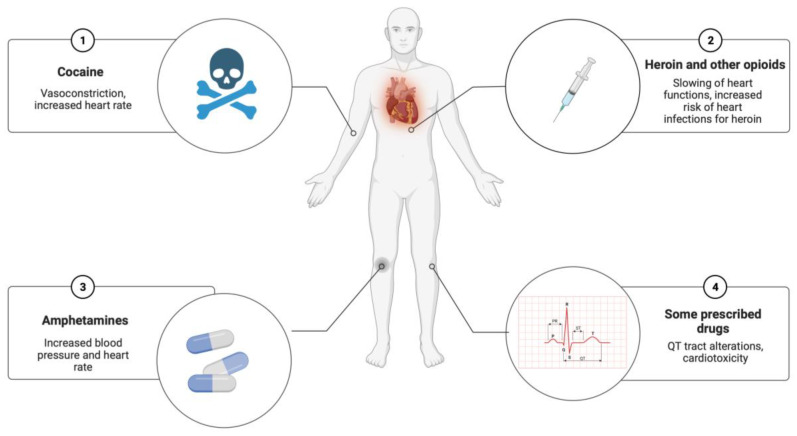
Exogenous substances as potential triggers in SCD (created with BioRender.com).

The role of exogenous substances, such as illicit drugs or prescribed medications taken before death, as triggers of SCD should not be underestimated (Figure 4). The literature has provided several studies on the cardiotoxicity of illicit narcotic substances. For example, cocaine can quadruple the risk of SCD, inducing vasoconstriction, increased heart rate, and increased blood pressure, with reduction of blood flow to the heart. Heroin can cause coronary vasospasm and abrupt hypotension with acute myocardial ischemia. Furthermore, the role of new illicit drugs from the street market must be considered, requiring close collaboration with expert toxicologists [7,11]. Various prescribed drugs, such as antipsychotics, can also cause cardiotoxicity, such as myocarditis or QT alterations.

### 3.7. Advantages in the Use of Proteome Analysis in the Post Mortem

The review showed that most of the studies have focused on the context of the dosage of markers of cardiac damage, especially in ischemic or coronary diseases, through protein analysis methods such as immunoassays. Fewer studies to date have analyzed the human cadaveric proteome in the context of SCD using more complex technical methodologies, such as mass spectrometry. This may be related to the cost and complexity of carrying out these investigations. However, the review demonstrates the benefit that a proteome study can offer in diagnosing SCD mechanisms and related etiologies, understanding pathophysiology, and defining promising biomarkers.

### 3.8. Existing Limitations in Forensic Pathology

The review also showed the presence of several limitations in the use of proteins in the post mortem, especially as regards the dosage of markers of cardiac damage.

The first limitation is related to conflicting results between the various studies. For example, in the analysis of cTNT, some authors did not find a significant increase in cardiac blood in the SCD group compared to the control group [50]. Other authors did not find a significant difference in pericardial fluid for the marker cTnI in the two groups compared, reporting that troponin levels and other damage markers can also rise in other cases, such as in asphyxiation [51]. We believe that these elevation mechanisms are associated with terminal hypoxic processes in the agonic phase, as well as with the possibility of terminal myocardial ischemia in various non-cardiac causes of death. For example, in asphyxias associated with compression of the nervous vascular bundle of the neck, it is known that there are mechanisms of action on the vagus nerve that can induce bradycardia reflexes on the heart with fatal arrhythmias. The mechanisms of terminal myocardial injury can also occur in other causes of death, such as hyperthermia, drug abuse with cardiogenic effects, and cerebrovascular disease, where it is possible that there may have been mechanisms of terminal cardiac injury [52].

According to other authors, peripheral blood does not always provide consistent results. For example, according to the study by Lai et al., the cTnT levels in cardiac deaths were no higher than those in the control group in peripheral blood. In addition, it is necessary to consider the possibility of a greater release of markers into the circulation in cases of traumatic cardiac injuries (bruises, lacerations, etc.) [53].

It should also be considered that post-mortem samples, especially those examined several days after death, may show high levels of hemolysis. Post-mortem changes related to putrefaction and post-mortem proteolysis should be evaluated as potential variables to be analyzed in cadaveric proteome studies. Rahimi et al. evaluated the cTnT protein levels in post-mortem serum by studying a total sample of 118 cases. The analysis showed that there was no correlation in the different groups examined on post-mortem serum [54].

Hemolytic mechanisms in the sample may result in elevation of markers, as the free hemoglobin component may interfere with the assay. In several studies, a portion of the samples was excluded from the analysis because the sample showed severe hemolysis. In order to overcome this limit, investigations should be carried out immediately, avoiding refrigeration and thawing cycles, and subjected to preventive and accurate centrifugation.

**Figure 5:**
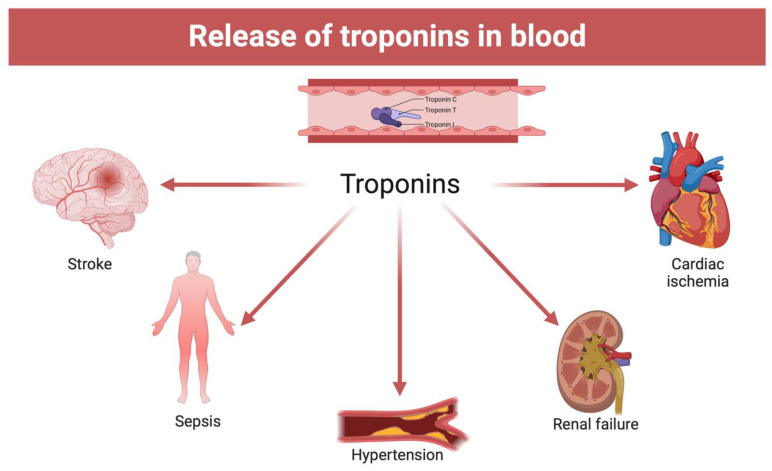
Alternative non-cardiac causes of troponin blood elevation (created with BioRender.com).

However, the previously described studies disagree with respect to others that have provided positive results for the analysis of cardiac troponins in peripheral blood [55,56]. Such discrepancies could require greater uniformity in the methods of conservation of the body, collection of the biological sample, pre-treatment of the sample before analysis (centrifugation and possible dilution), and methodology of analytical investigation. Another important explanation concerns the choice of the sample, also considering the role that alternative non-cardiac pathologies can have, such as pulmonary embolism, pulmonary edema, renal failure, pulmonary hypertension, sepsis, and septic shock (Figure 5). In this regard, the pericardial fluid is the one that today would have provided the best results with better sensitivity than other samples in the context of the dosage of markers of cardiac damage.

Another explanation of the discrepancies would concern the sensitivity of the chosen markers, signaling that in processes of acute ischemia, there may not be an immediate elevation of cardiac markers. In this context, the analysis of highly sensitive markers is preferable. Another limitation is related to the often limited number of samples examined in the studies cited. This limit could be related to the difficulty in collecting biological samples from human cadavers for experimental analyses, but also to the costs related to proteomic investigations. The advantage of proteomic investigations in these cases is represented by the possibility of having laboratory data useful to support autopsy findings, especially in cases in which standard post-mortem investigations have not identified pathognomonic cardiac injuries. The disadvantage could be determined by the presence of intrinsic and extrinsic variables that could affect the proteome, including the standing temperature of the corpse, the humidity, and the post-mortem interval (PMI). A correct and cautious interpretation of post-mortem proteome investigations is essential [57]. This interpretation cannot disregard the collection of clinical anamnestic data by examination of signs and symptoms, the visualization of ECG, or laboratory instrumental investigations, if available. Furthermore, an accurate analysis of comorbidities is necessary, especially when proceeding with the dosage of post-mortem cardiac markers, which can be elevated in other pathologies. Evaluation of cases of sudden cardiac death always requires an autopsy, visualization of the severity of coronary artery disease, and evaluation of the specific case. The definition of reference cut-off, as in the clinic, for cardiac markers still remains very complicated due to intervention of post-mortem alterations, which could increase with the progression of the PMI and the degree of hemolysis of the sample, if not promptly analyzed. This degree could increase if the sample is refrigerated for a long time at low temperatures; therefore, prompt analysis of the biological matrix is recommended.

## 4. Conclusions

Post-mortem biochemistry through the study of the proteome is certainly promising as a supportive tool in forensic investigations and aims to improve and reach ever higher levels of diagnostic sensitivity and specificity. Now, the evolution of scientific research and the increased accuracy of various disciplines will be oriented towards the elaboration of an “algorithm,” able to weigh the value of “evidence” placed at the disposal of the “justice system” as real truth and proof [58]. Current evidence in the literature suggests that proteome analysis can be very useful, especially in cases where autopsy and histopathological investigations are not sufficient. The application of these methods always requires the execution of all traditional post-mortem investigations. The future perspectives of this discipline should aim at the creation of multiparametric panels of markers applicable to different biological matrices such as pericardial fluid, cardiac blood, and peripheral blood. The latter should represent the ideal sample when a simple external cadaveric examination is carried out, as often happens in cases where the judicial authority does not order an autopsy. However, pericardial fluid seems to show better sensitivity and specificity for cardiac marker analysis. In this direction, validation of diagnostic tests is necessary with the definition of standard and uniform methods among forensic pathologists. Further proteomic studies are desirable, especially to search for protein biomarkers of ventricular arrhythmias and structural cardiac alterations, which are still scarce in human models.
